# Virtual OSCE Delivery and Quality Assurance During a Pandemic: Implications for the Future

**DOI:** 10.3389/fmed.2022.844884

**Published:** 2022-04-04

**Authors:** Shannon L. Saad, Cassandra Richmond, Karina Jones, Michelle Schlipalius, Helen Rienits, Bunmi S. Malau-Aduli

**Affiliations:** ^1^School of Medicine, Notre Dame University, Sydney, NSW, Australia; ^2^College of Medicine and Dentistry, James Cook University, Townsville, QLD, Australia; ^3^School of Medicine, Monash University, Melbourne, VIC, Australia; ^4^School of Medicine, University of Wollongong, Wollongong, NSW, Australia

**Keywords:** virtual OSCE, quality assurance, pandemic/COVID-19, Quality framework, clinical assessment

## Abstract

**Background:**

During 2020, the COVID-19 pandemic caused worldwide disruption to the delivery of clinical assessments, requiring medicals schools to rapidly adjust their design of established tools. Derived from the traditional face-to-face Objective Structured Clinical Examination (OSCE), the virtual OSCE (vOSCE) was delivered online, using a range of school-dependent designs. The quality of these new formats was evaluated remotely through virtual quality assurance (vQA). This study synthesizes the vOSCE and vQA experiences of stakeholders from participating Australian medical schools based on a Quality framework.

**Methods:**

This study utilized a descriptive phenomenological qualitative design. Focus group discussions (FGD) were held with 23 stakeholders, including examiners, academics, simulated patients, professional staff, students and quality assurance examiners. The data was analyzed using a theory-driven conceptual Quality framework.

**Results:**

The vOSCE was perceived as a relatively fit-for purpose assessment during pandemic physical distancing mandates. Additionally, the vOSCE was identified as being value-for-money and was noted to provide procedural benefits which lead to an enhanced experience for those involved. However, despite being largely delivered fault-free, the current designs are considered limited in the scope of skills they can assess, and thus do not meet the established quality of the traditional OSCE.

**Conclusions:**

Whilst virtual clinical assessments are limited in their scope of assessing clinical competency when compared with the traditional OSCE, their integration into programs of assessment does, in fact, have significant potential. Scholarly review of stakeholder experiences has elucidated quality aspects that can inform iterative improvements to the design and implementation of future vOSCEs.

## Introduction

Pandemic disruptions to medical education have driven innovation in relation to the delivery of assessment tasks. From a clinical assessment perspective, performance is often evaluated through the Objective Structured Clinical Examination (OSCE) (1). The traditional OSCE is a standardized method for assessment of clinical competence across a range of domains, including communication, procedural and practical skills. The traditional OSCE requires an examiner to observe the candidate's performance across these domains, and therefore cannot simply be replaced with knowledge tests or oral vivas. However, this form of assessment has typically relied on the congregation of large numbers of people, including students/trainees, examiners, simulated patients (SPs), invigilators and facilitators (2). During the COVID-19 pandemic, this traditional face-to-face OSCE format presented a contravention of the physical distancing requirements put in place to mitigate viral infection. Although some researchers have reported the possibility of conducting OSCEs with the best infection prevention measures in place (3), many medical schools were forced to rapidly develop an adapted OSCE format (4–6). The modified approaches adopted had a range of underlying designs and, although complex, have aided adherence to physical distancing requirements through online measures of clinical performance assessment, with examiners, students/trainees and standardized patients' video-linked into the OSCE (4, 5). Remote OSCE observation via videoconferencing has been successfully used to research differences in assessor scoring behaviors, showing feasibility of the use of virtual formats (7). Similar approaches have been used for bespoke high stakes postgraduate examinations (8).

In the Australian and New Zealand context, the impact of the COVID-19 pandemic varied between regions, however the uncertainty of the pandemic forced medical educators to develop OSCE formats that could function in the event of increased restrictions. The Australasian Collaboration for Clinical Assessment in Medicine (ACCLAiM) is a voluntary assessment consortium, involving 14 medical schools across Australia and New Zealand. The aims of ACCLAiM are to benchmark medical student clinical assessment outcomes, and to provide QA reports for exit-level OSCEs (9). The ACCLAiM QA process involves a QA visitor from one member school attending, and observing all aspects of, the graduation-level OSCE of a second member institution. Following the visit, the QA visitor completes a structured report aimed at quality improvement (10). The collaborative QA process within ACCLAiM has fostered a community of practice in clinical assessment, with professional development, shared resources and scope to optimize standards in OSCE processes being key benefits (9, 10). During 2020, most of the ACCLAiM QA visits were conducted in remote (vQA) formats. As such, the ACCLAiM QA process provides an opportunity to investigate the Australian experience of vOSCE and vQA during a pandemic.

Given the widespread impact of the COVID-19 pandemic and possible future occurrence of global disruptions, it is relevant to evaluate the quality and utility of these formats of assessment in the context of a pandemic and the associated quality assurance (QA) processes to derive lessons for potential future applications. Fuller et al. (11) advised that collecting evidence of both intended and unintended consequences of assessment change, and the views of the students/trainees, patients (real and/or simulated), and administrators will be vital. Researchers have examined the impact of COVID-19 on the medical curriculum and competency-based assessment within the clinical learning environment (11–16). However, there is a paucity of literature on the exploration of the experiences of the multiple stakeholders who have been involved in the new virtual OSCE (vOSCE) formats.

Therefore, this study aimed to explore the experiences of all stakeholders hosting or attending the vOSCEs at ACCLAiM member schools. Stakeholder perspectives regarding the future implications for vQA of clinical assessment activities and their recommendations for the quality implementation of vOSCE formats were also investigated. Harvey and Green's (17) validated Quality Framework was employed to explore stakeholders' experiences and perspectives, and to evaluate the quality and utility of the vOSCE and vQA formats during pandemic conditions.

### Theoretical Framework

Evaluation of the quality of the vOSCE formats is crucial to ensure they retain the ability to derive robust information for fair and accurate measurement of student clinical performances. Assessments should ensure the achievement of learning outcomes and the acquisition of the competencies required for safe practice drawing on the elements of utility described by van der Vleuten, namely reliability, validity, educational impact, acceptability and feasibility (18). Encompassing these principles is the notion of quality in the delivery of higher education which can be contextualized within five possible definitions: quality as exceptional; quality as perfection; quality as value for money; quality as fit for purpose; and quality as transformative (17, 19). Within the context of the vOSCE, quality must be considered from all stakeholders' viewpoints, including the students/trainee, examiner, SP, QA visitor, and administrator. In this study, we define quality as exceptional to indicate that the vOSCE fulfills the high standards expected of a traditional OSCE exam. Quality as perfection refers to the extent to which the vOSCE conforms to specifications, whilst avoiding defects. Quality as transformative means that changes made to the examination process enhanced the experience for those involved. Quality as fitness for purpose considers if the vOSCE and/or vQA fulfills the explicit objectives and mission of stakeholders. Finally, quality as value for money assesses if the process is cost effective (17). Utilizing a theory-based approach to explore stakeholder experiences strengthens the transferability of any findings. This results in greater opportunities for developing improvements to virtual clinical assessment methods.

## Methods

### Study Design

This study utilized a descriptive phenomenological qualitative design, which aims to describe the essence of an experience, focusing on what is essential and meaningful (20). Focus group discussions (FGDs) were chosen to facilitate discussion and interactions within a nurturing environment (21), to explore the experiences and perspectives of all stakeholders through the lens of QA and utility of the new assessment format and were conducted online using the Zoom videoconferencing platform. The use of virtual focus groups in qualitative research was necessitated during the global pandemic, with good feasibility and acceptability (22, 23). To this end, our study utilized a virtual approach for the focus group discussions.

The focus group questions were collaboratively developed by the researchers to align with Harvey and Green's (17) Quality Framework, as described above. This framework was also used in the concept-driven analysis of the data. The concept-driven analysis involved iterative stages of labeling, classifying and organizing data into main themes, concepts and categories in a theoretical framework (24). This method fostered understanding of participants' views and experiences in relation to the utility of the vOSCE and vQA. This work was conducted under Permit H6833, granted by the James Cook University Human Research Ethics Committee.

### Study Setting, Context, and Participants

An email invitation was sent to the academic assessment leads at each of the four medical schools within the ACCLAiM collaboration that utilized vOSCE and vQA during the study period. All four schools accepted to participate, but one school withdrew when their OSCE was canceled due to a snap lockdown. Individual participants were purposively sampled by invitation from the academic assessment leads at the three participating medical schools. Prospective participants from all stakeholder groups that were involved in vOSCE in 2020 and received ACCLAiM vQA were invited by email. Additional email invitations were sent to ACCLAiM academics from other medical schools who served as ACCLAiM vQA visitors for the study vOSCEs. All invitations included a detailed information sheet about the study.

### Data Collection

The FGDs were conducted between November and December 2020 using online video-conferencing programs. The focus groups were held no more than 4 weeks following the vOSCE, with most being held within a week. The participants included students, vOSCE station examiners, vQA visitors, simulated patients, and involved professional staff/administrators at three ACCLAiM participating medical schools. Verbal consent was obtained from each participant prior to the commencement of discussion. The FGDs were organized by KJ and conducted by BMA, and continued until data saturation was achieved. Each FGD lasted between 40 and 60 min, and were audio recorded. The discussions were based on semi-structured, open-ended questions, based on the theory of quality as described by Harvey and Green (17)—see Appendix 1 for the focus group discussion guide.

### Data Analysis

An external transcription service was engaged to transcribe the audio-recordings from the FGDs. Two members of the research team (SS and CR) conducted concept-driven framework analyses, using Harvey and Green's (17) Quality Framework. This approach combines inductive and deductive analytical procedures based on the five stages of framework analyses as outlined by Ritchie and Spencer (24): familiarization, identifying a framework, indexing, charting, mapping and interpretation. This involved deductively coding informational meaning units to the elements of the QA theory, and further developing subcategories within these categories using an inductive approach (24). Final consensus regarding the coding was settled by iterative discussions involving the whole research team. The identified themes are presented using illustrative quotes that are affixed with the participant's host school, individual participant number, gender and stakeholder role. For example, participant S1-P6-M-St refers to School 1, Participant 6, Male, Student.

## Results

The vOSCEs were conducted on the Zoom online platform. However, the design differed at each of the participating medical schools. Table 1 describes the architectures used. Five FGDs were held, involving 23 participants from five medical schools and representing a broad range of stakeholder groups who experienced a vOSCE in 2020 (see Table 2). Data saturation for the major themes, described below, occurred after the fifth FGD, where the perspectives of the vQA visitors were elucidated.

**Table 1:** vOSCE designs used by the participating medical schools in 2020.

**vOSCE features**	**School 1**	**School 2**	**School 3**
Location of students	Off-campus	On-campus	Off-campus
Location of examiners	Off-campus	Blend of: • on-campus co-located with student • off-campus	On-campus - co-located with SP
Location of SPs	Off-campus	Blend of on-campus, separated off-campus	On-campus - co-located with examiner
Location of QA	Off-campus ACCLAiM vQA and internal academic staff member vQA	Off-campus ACCLAiM vQA	Off-campus ACCLAiM vQA and internal academic staff member vQA
Location of invigilators	Off-campus	On-campus	On-campus - separate hub from SPs/examiners
Provision of stems	Released in advance	Not released in advance	Not released in advance
Number of stations	Eight	Eight	Six
Station Time	Eight minutes	Eight minutes	Twelve minutes

**Table 2:** FGDs participant characteristics^*^.

**FGD**	**Participant characteristics**
1	2 × FPr; 1 × FAc/QA; 1 × FAc/Ex; 2 × MSt; 1 × FSP
2	2 × FPr; 1 × MPr; 3 × FEx
3	1 × ME × ; 2 × FPr
4	1 × MSP; 1 × FSP
5	4 × FQA; 1 × MQA

* *F, Female; M, Male; Pr, professional staff member; Ac, Academic; QA, Quality assurance visitor; St, student; SP, Simulated patient; Ex, Examiner*.

All participants indicated that the vOSCE was necessitated by the pandemic—“it was the assessment we had to have”—and was rapidly developed by schools to optimize robustness of clinical assessments in the context of physical distancing requirements. Participants' perceptions were explored through the five elements of Harvey and Green's (17) Quality Framework.

### Quality as Exceptional (Fulfills the Basic Minimum Standard of an OSCE Assessment)

In terms of assessing history-taking and communication skills alone, the vOSCE met the basic minimum standard of an OSCE assessment when compared with the traditional format, which is considered the “gold standard” in OSCE delivery.

“*The internet connection was fine. And the students just did their history and their communication skills as they would in a normal OSCE.”* S3-P14-F-Pr

“*In terms of interacting with the patients, I think [the vOSCE] was quite positive, and very resemblant of what we'd come to expect with in-person history-taking.”* S1-P7-M-St

However, some stakeholders noted that the vOSCE had limited ability to assess other important clinical skills, such as physical examination and procedural skills, that would otherwise be assessed in a traditional OSCE. In this regard, it appears that the vOSCE format restricted the ability to assess the full range of clinical skills that would typically be assessed in a traditional OSCE, and therefore does not meet the basic minimum standard for this type of clinical assessment.

“*The limitations of the [vOSCE] are that we can perhaps only assess 50% of the range of skills that we need to assess for a graduating student. If we can't see their procedural skills, and their physical examination skills... we are talking about significant limitations of [vOSCE] as an exit exam.”* S2-P8-F-Ex

In addition, the assessment of some aspects of a communication skills domain in the vOSCE were restricted due to lack of direct interaction between the student and the simulated patient.

“*I think … you're limited in your assessment of rapport building, because it's difficult to build rapport over an online platform. And it's more difficult for an examiner to then see that body language interaction.”* S2-P11-F-Ex

Although the vOSCE fulfilled the basic minimum standard of an OSCE assessment when assessing some clinical skills (such as history-taking), it was limited in its overall ability to assess the full range of clinical skills that are typically assessed in a traditional OSCE. In this regard, the vOSCE could not be described as exceptional in quality as a stand-alone clinical assessment activity.

### Quality as Perfection (Conforms to Specifications, Whilst Avoiding Defects)

The relatively untested nature of the vOSCE led to the anticipation of technical difficulties that could affect the quality of the assessment and was likely to be related to the participants' confidence and experience of videoconferencing.

“*So, I was nervous about technology. I anticipated disaster.”* S2-P8-F-Ex

“*My expectation was more around human error. …Maybe I might have sent the wrong information, or the SPs access the wrong information... I believed in the technology… we've used Zoom quite a lot…so I trusted the technology.”* S2-P9-M-Pr

Due to the novelty of the new format, a wide variety of strategies were employed to minimize errors. These included pre-briefings, stakeholder training, extra in-station professional supports, practice exam sessions, real-time communication through online messaging platforms (such as WhatsApp), and expert input to support the information technology required to run a vOSCE.

“*I had every confidence in the planning side and the training and the preparation, that sort of administrative side of things… because that can be prepared and double checked over and over before the day.”* S2-P13-F-Pr

“*The good thing about having… a concierge for each room, while I guess it uses more staff… it just means the examinee, the examiner and the SP don't have to worry about that stuff. And we know someone else is keeping [the] timing … that was very helpful.”* S3-P17-F-SP

The stability of the internet connection for a vOSCE (i.e., where participants were not on campus and access to IT technicians was limited) was noted to be high-risk, and this was more difficult to control or plan for than aspects such as participant training.

“*I think the challenge sometimes is the connection. So for example, in my station, my student actually kind of [froze] for a few seconds… so she must have… experienced some… difficulties with her internet.”* S3-P15-F-Pr

“*I think sometimes if you had a bad connection, that lag that you sometimes get, I had to really watch that I wasn't talking over students when they're asking questions or giving me information.”* S1-P4-F-SP

Participants reported overall satisfaction with how the vOSCE conformed to their expectations, and the level of errors experienced.

“*From a student perspective… for me personally… it went really smoothly.”* S1-P6-M-St

“*I thought it exceeded my expectations as to how well it all worked, and how well it fit together and how very few issues we had. We really didn't have anything come up that was... any sort of catastrophe.”* S2-P9-M-Pr

For vQA, the aim was to maximize experience for the vQA visitor, with a thorough pre-briefing on the locally used model and how to navigate engagement with the system. This was found to be essential for the observation and feedback process.

“*So I got very used to the electronic format very early on, I think because the briefing had been good.”* S4-P21-F-QA

Despite technical and procedural issues being anticipated by all stakeholders due to its novel format, the vOSCE ran relatively uneventfully. In this regard it may be considered as possessing features of perfection as a marker of quality.

### Quality as Transformative (the New Processes Enhanced the Experience for the Participants)

Although the recording of OSCE performances is not a novel concept, the vOSCE format has in-built functionality in this regard; therefore, adapting to an online delivery format meant that student performances at vOSCE stations could be easily recorded. This was perceived to be a beneficial outcome for both the students and the school in cases where fairness or allocation of grading was being challenged.

“*You have the ability of recording the whole session, and in case the student challenges the exam, they see everything is recorded. So, if they challenged…you can always go back and have it double marked.”* S3-P16-M-Ex

“*[As it's] recorded [it] means that [if the] student… ends up failing a station, we can get it reviewed.”* S1-P6-M-St

Furthermore, due to the online delivery of the vOSCE, professional staff performing online invigilation reported that they had access to parts of the clinical assessment that they would not normally have experienced in a traditional OSCE. This was perceived to be beneficial as it provided new insights for key stakeholders regarding the impact of the OSCE experience on students.

“*In my role as an invigilator, I don't usually go inside the stations to actually listen to the students' exam performance. So, this is the first time I'm actually inside a room with the students. So, I finally get to see…why some of them are very stressed, why some, occasionally will come out with tears in their eyes.”* S3-P15-F-Pr

From the SP perspective, the ability to hold the “patient script”, whilst keeping it out of view, was identified as a benefit of having the vOSCE format. In this regard, some SPs felt that being able to refer to the script during the assessment ensured consistency of the simulation for each student, that is an important feature of a standardized delivery.

“*The other thing I absolutely loved was I could have my script in front of me. And I would be completely consistent with the opening line. So…it was really fantastic*.” S1-P4-F-SP

However, some SPs noted that, due to the rigid structure of the vOSCE, the ability of the SP to interact with the examiner in between students was limited. This meant that some felt less supported during the vOSCE as they were unable to receive examiner feedback about their performance in real time.

“*I missed a little bit of the chitchat in between candidates with the examiner and also I… like to calibrate with those first couple of students my performance [in case] I've missed anything.”* S1-P4-F-SP

From the QA visitor's viewpoint, the inability to network with members of the host school was viewed to be a detraction of the vOSCE.

“*You didn't have that opportunity to socialise and to network with the school that you were visiting, which is something that you certainly can do when you attend face-to-face. So that was probably something that was missing*.” S3-P19-F-QA

The vOSCE was deemed to enhance the experience for stakeholders involved in ways that had direct impact on actual the running of the clinical assessment itself. In this regard, the vOSCE is considered to possess transformative qualities.

### Quality as Fit for Purpose (Fulfills the Explicit Objectives and Mission of Stakeholders)

Pandemic disruptions necessitated rapid and innovative changes to clinical assessments. Within the context of the physical distancing requirements of 2020, the vOSCE enabled schools to proceed with familiar processes with adaptations suited to the mandated conditions.

“*All the changes in placement, the…changes in curriculum, everything that's happened this year, I think that these were the perfect assessment for us*.” S1-P7-M-St

“*From our perspective, completely fit for purpose. You know, things were proceeding exactly as they would, if it was live and in person… I thought we were achieving what we set out to do.”* S2-P13-F-Pr

Similarly, despite travel restrictions and local lockdowns, the new mode of delivery of clinical assessments meant QA visits could proceed remotely.

“*I thought it was really useful. I thought it was great that we could still do quality assurance, even though we couldn't travel as freely*.” S3-P19-F-QA

As an overarching objective in 2020, schools needed to be able to make fair and robust decisions about student skills attainment to allow progression to internship, and the vOSCE facilitated this process.

“*I think for this year, the purpose of the [vOSCE] has shifted away from the traditional OSCE. It is a summative exam, no doubt. But… our main aim is just to see whether… the students reached the minimum competence expected... We just want to know whether they pass or fail overall. [In] that regard, I think you have achieved part of the aim of the [vOSCE] itself.”* S1-P2-F-Ex

Where it was decided to award marks to vOSCE performance, the spread of student performances was consistent with past marks, implying that, on this limited evidence, the instrument was measuring what it was meant to measure despite its novelty.

“*From the academic point of view, it hasn't been any different at all. It's given us the same spread of results, which means that the assessors all adjusted, obviously, and… the actual ranking of the students has been as predicted. It didn't change that, just because we went to a [vOSCE].”* S2-P12-F-Ex

While participants expressed concern about the ability of the vOSCE to meet minimum standards in the assessment of some skills (see Quality as Exceptional), consideration was given as to how the vOSCE, as an assessment of clinical competence, could be included in the design of a wider program of assessments.

“*Say for example, you want to get a student to talk through what they would do in a case. Then at least you know I haven't assessed… ‘does', I've only assessed ‘knows how'. I'm down one on Miller's Pyramid. If you acknowledge that's what you've done…if you're mindful of what am I testing and to what level, then it's kind of okay.”* S3-P20-F-QA

“*We couldn't assess the actual hands-on physical examination, but we could certainly assess skills around it. Noting that we are looking at final year students who should have been assessed in their physical examination technique at other points throughout their course. Looking at their …clinical reasoning, how they would investigate, diagnose and [make] diagnostic formulations, and management plans*.” S3-P19-F-QA

Changes to the delivery of medical care during the pandemic, with the introduction of telehealth (in particular virtual medical care) and the need for medical practitioners to adapt their skills to the virtual environment, was aligned with using the vOSCE as an assessment activity.

“*I think telehealth is here to stay even after COVID and that's quite well suited to that [vOSCE] format.”* S5-P22-M-QA

“*The OSCEs this year prove the point that the good students aren't just academically good, or just [have] good communication skills, but they're able to adapt and be flexible. And obviously, they need that in a clinical environment.”* S2-P11-F-Ex

One significant concern regarding vOSCE suitability was the inability to enforce exam conditions in some of the formats leaving students without strict invigilation, with the possible consequence of breaches in examination security and consequent academic misconduct.

“*The biggest issue we have was the exam integrity, because you can't sequester your students, and you can't examine them all at exactly the same time, you can do it within a short period of time, you can't do it all at exactly the same time. So, I think exam security is a huge potential issue.”* S1-P22-F-QA

A further concern expressed is the unintended impact of the new formats on student learning, by encouraging students away from face-to-face learning in the clinical environment.

“*The traditional OSCE drives [students] to get together in groups and in pairs, it drives them to see patients to practice their examination skills. I just worried that the [vOSCE]doesn't necessarily drive the learning as well.”* S1-P5-F-Ac/Ex

vQA was found to have the benefit of less obvious disruption to the examination process, and thus increased suitability to an activity that relies on observation.

“*I had the video off [and] microphone off. And I think my title just said QA. So, I was literally invisible. And I knew that I wasn't impacting on anything So, in some ways, I actually felt more connected because I could just focus on what I was seeing and hearing not on, ‘How do I get out of this room quickly? And how do I get into the next room?”'* S4-P21-F-QA

“*I really did feel like a fly on the wall. I felt like a bit of a spy…I thought we really didn't disrupt the process as much. Once you're in and your video was off, it was wonderful.”* S3-P20-F-QA

Noting the physical distancing restrictions during peak pandemic conditions, the vOSCE was able to be delivered, and was deemed to be relatively fit for purpose as a marker of quality.

### Quality as Value for Money (Is Cost Effective)

Due to 2020 pandemic disruptions, medical schools had to change to online delivery of teaching, with the result that most of the hardware and software required for vOSCE delivery was already available. Instead, the real costs were found in the time staff spent designing and testing vOSCE formats to ensure dependable delivery of the assessment.

“*We already had the computers, we had the laptops, we had to invest in some more webcams. So the cost wasn't high. I think the cost was high in terms of time… of how much time it costs the assessment team to find an applicable way to run it*.” S3-P14-F-Pr

“*The cost was not greatly increased, because in most cases, we used permanent staff, although there was a significant workload increase for those. And it really did rely heavily on technology, but everyone these days seems to have their own laptop. So that seemed to be okay*.” S1-P3-F-Pr

vOSCE architectures that relied on additional staff to oversee technical aspects of the assessment, and thus free up students, assessors and SPs to participate without the stress of navigating technology, incurred additional staffing burdens.

“*The model that we've chosen is very heavily reliant on administrators. So, we recruited clinical site administrators that are employed by the hospitals. And we trained them on Zoom and trained them how to run the meetings. So that was about 80 people in total… I also employed about five casual staff, so that staff could have a break.”* S1-P3-F-PR

Some participants had the overall impression that in comparison to traditional OSCE delivery, there would be cost savings due to simplification of running the assessment remotely.

“*I think the [vOSCE] format works really well…I'm really interested to know is this the way of the future? Because in some ways, there's a lot of simplicity about it. There's a lot of saving of resources.”* S4-P21-F-QA

In particular, vQA visits between schools in different states were perceived to be a cost-effective enterprise, and a responsible alternative for a higher-education sector impacted economically by the pandemic.

“*And the added benefits are really you don't have to travel…obviously in a pandemic situation...The cost is massive currently for universities, the environmental aspect, and just the time aspect.”* S3-P19-F-QA

**Figure 1:**
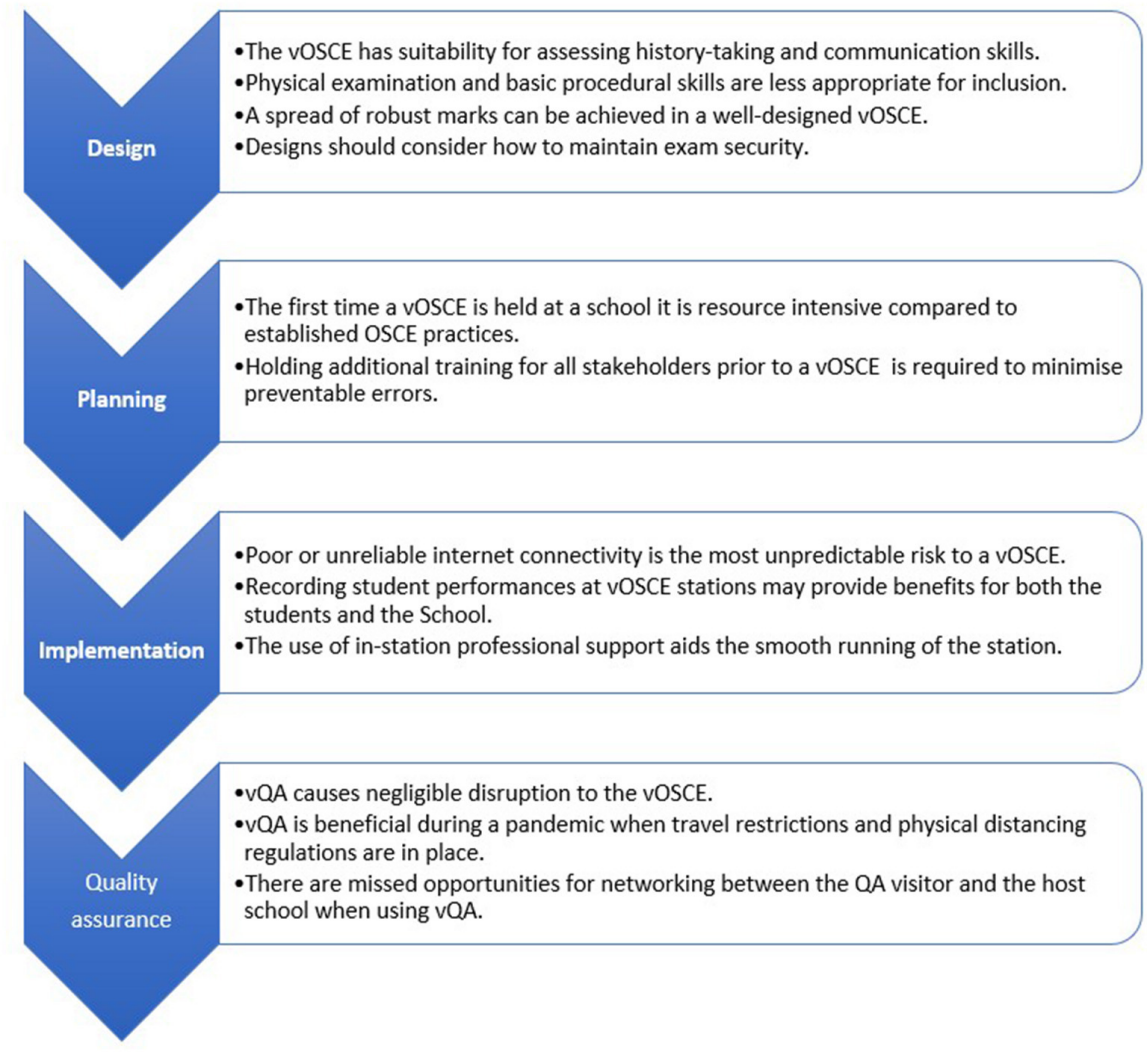
vOSCE 2020 lessons learned.

Overall, Figure 1 provides a summary of what was gleaned from the participants' perspectives during vOSCE and vQA implementation. While the vOSCE enabled standardized assessment of student clinical performance, there were limitations compared to the traditional OSCE. The transition to virtual delivery is onerous and requires intensive planning, and training of all stakeholders to ensure minimal errors.

Although considerable time and effort went into the planning of the virtual OSCE (particularly given the novel status of this clinical assessment format), the running of the day itself was relatively inexpensive. This is because the vOSCE did not require bringing together (and paying) the usual large groups of people involved (including a range of actors and invigilators), and other associated costs (including travel and catering). In this regard, the vOSCE is considered to be value for money.

## Discussion

Uniquely, this study has used a Quality framework to explore stakeholder perspectives regarding the virtual delivery of clinical assessments. Necessitated by the pandemic in 2020, medical schools rapidly innovated to accommodate the need to deliver robust and defensible assessment of student performances in the context of physical distancing requirements. In exploring the diversity of participants' experiences of the vOSCE and vQA, the assessments we “had-to-have” appear to be appropriate for 2020 pandemic circumstances, with greater understanding gleaned regarding the limitations of the vOSCE compared to a traditional OSCE. In relation to whether the vOSCE is fit for purpose, it is reassuring that assessment of several key competencies (history taking, communication skills and clinical reasoning) were relatively suited to the online delivery format. However, in contrast, to assessment of physical examination and procedural skills, could not be achieved through the vOSCE designs used by participating schools. With the ongoing growth of telehealth services, vOSCEs could gain further validity as an assessment tool when used as a tool to assess virtual medicine competencies. Technology-based assessments and vQA have the potential to make the process of administration more standardized and efficient as well as cost effective (quality as value for money) and this may be a major strength of this new approach.

While benefits were identified, the limitations and drawbacks of online clinical assessments remain. The most virtual formats, where examiners, students and simulated patients were all online, had the greatest impact to the quality of the vOSCE in meeting the minimum acceptable standard of a clinical assessment (quality as exceptional); and significant preparation and upskilling is necessary for the prevention of technical errors (quality as perfection). The finding that the vOSCE had novel benefits (quality as transformative) was balanced by the preference expressed by all stakeholders for the traditional format as a “gold standard” clinical assessment. This finding agrees with reports from published evaluations conducted on vOSCEs in other medical schools (4, 5). Studies have highlighted that face-to-face collaboration in quality assurance processes fosters a community of practice in clinical assessment, providing networking and professional development opportunities (10). Though opportunities to network were perceived to be reduced through vQA, this format was reported as not only being feasible, but also as having additional potential benefits when compared with in-person QA e.g., vQA participants could observe the assessment from a relatively concealed viewpoint, with less risk of disturbing the assessment and possibly less impact on the processes observed than face-to-face QA. These perceived benefits warrant further investigation of the wider utility of vQA, specifically for face-to-face clinical assessment formats.

Validity concerns regarding virtual OSCEs have been expressed (25), and justifiably highlight that these formats require rigorous review when considering future implementation. Aligned with these concerns, we did find evidence of the following threats to validity: altered non-verbal communication and poor utility for assessment of physical examinations and procedures, that could decrease generalization of assessment outcomes to clinical encounters. There were also difficulties establishing examination security, and evidence of altered student preparation for vOSCEs with implications for undesirable educational influences. While one participating school mentioned a reassuring spread of marks generated by their vOSCE (when compared to previous OSCE performances in earlier years), further research taking a detailed and rigorous approach is required to provide convincing evidence of vOSCE validity. Care was also taken with vOSCE designs to minimize technology-created cognitive load on examiners and video-conferencing fatigue on all participants that could impact standardization and scoring. Nonetheless, researchers have reported that validity evidence needs to go beyond a high Cronbach's alpha value or generalisability coefficient (26). In addition to psychometric properties, educational impact, practicability, cost-effectiveness and acceptability also contribute to the utility value of assessments (18). From a practical viewpoint, feasibility issues with vOSCEs and vQA were related to availability of internet connectivity, time and human resources necessary for the smooth running of the assessment.

This study contributes alongside that of other health professions researchers publishing their experiences during the COVID-19 pandemic. Where other studies have focussed on important practical considerations based on their pandemic experiences (5, 6, 27, 28), we have used a Quality framework to examine the utility of the new practices. In synthesizing the experiences of our participant schools, we engage in reflective practice (29) to achieve deeper understanding of the practical utility and quality indicators of the vOSCE and enable iterative improvements in the field. The lessons learned could serve to guide further developments in relation to the vOSCE, with major considerations particularly around issues concerning security, resourcing and training for all stakeholders.

## Strengths and Limitations of This Study

This study employed a theoretically informed qualitative research approach with purposive sampling of stakeholders involved with the vOSCE to provide an important synthesis of perspectives. The study adds to the literature regarding clinical assessment adaptations during the pandemic by providing recommended considerations for the quality implementation of vOSCE formats. However, it represents the views of only five Australian medical schools. Although there were a wide range of participants from different stakeholder groups, not all those involved in the vOSCE were interviewed, such as IT support staff and executive academics overseeing budgets. Additionally, there could have been participation bias - where those who agreed to be interviewed may represent views at either end of the spectrum. In this study, it was noted that both negative and positive responses were provided by participants, and all perspectives were explored through group discussions in order to minimize the impact of this possible bias. Finally, government restrictions and logistics unique to each school, meant that the architecture of each vOSCE was quite different, and thus comments were contextual depending on the school's vOSCE experience. Nonetheless, the detailed description of the study context and use of established theory suggests that the findings could be transferable to other settings with similar contexts.

## Conclusions

The impact of COVID-19 on medical education has presented the unique opportunity of trialing the vOSCE, which has functioned as a relatively “fit for purpose” tool in the remote assessment of a limited scope of clinical competencies of medical students—specifically history taking, clinical reasoning and potentially communication skills. The vQA process provides cost-effective and practical ways of assuring quality of clinical examinations and may enhance opportunities for remote quality assurance of face-to-face clinical examinations in addition to virtual formats. However, a validity argument for the vOSCE needs to be constructed with an examination of the evidence supporting the intended interpretations and uses of the assessment scores. Further studies on the impact of virtual clinical assessments on student learning would be useful to ensure scrutiny for unintended deleterious impacts. The effectiveness of the vQA process also deserves further research—particularly as we move to a “post COVID” world with ongoing travel restrictions and tighter institutional budgets.

## Data Availability Statement

The original contributions presented in the study are included in the article/Supplementary Material, further inquiries can be directed to the corresponding author/s.

## Ethics Statement

The studies involving human participants were reviewed and approved by the James Cook University Human Research Ethics Committee and conducted under Permit H6833. The patients/participants provided their verbal informed consent to participate in this study.

## Author Contributions

SS, CR, KJ, and BM-A conceived the study. BM-A and KJ conducted the focus groups. SS, CR, MS, and HR facilitated data collection. SS and CR analyzed the data and all authors advised on data analysis and interpretation. SS, CR, KJ, MS, HR, and BM-A contributed to writing the original draft. All authors reviewed, edited and accepted the final version of the manuscript.

## Conflict of Interest

The authors declare that the research was conducted in the absence of any commercial or financial relationships that could be construed as a potential conflict of interest.

## Publisher's Note

All claims expressed in this article are solely those of the authors and do not necessarily represent those of their affiliated organizations, or those of the publisher, the editors and the reviewers. Any product that may be evaluated in this article, or claim that may be made by its manufacturer, is not guaranteed or endorsed by the publisher.
