# Supplementary material for: Virtual OSCE Delivery and Quality Assurance During a Pandemic: Implications for the Future
Source: Front Med (Lausanne). 2022 Apr 4;9:844884. doi: 10.3389/fmed.2022.844884 (PMC9013903; doi:10.3389/fmed.2022.844884)
Supplement: Supplementary file 1 [file Data_Sheet_1.docx]

# Appendix 1 – Focus Group Discussion Guide

The aim of the research project was presented by the facilitator at the commencement of the focus group.

**Identifying the nature of quality:**

1. What was your overall experience of delivering the exit year OSCE as an online assessment?
2. What was your experience, if any, of the vQA process for the vOSCE?
3. What was your experience, if any, of the role of the vQA during the vOSCE?

**Quality as exceptional:**

1. What worked well in delivering the OSCE online?
2. Do you have confidence that the vOSCE was a valid tool for assessing the clinical competence of your final year students? How does the vOSCE compare to a face-to-face OSCE in terms of its ability to assess clinical competence of exit level students?
3. Do you have confidence that the vQA process at the vOSCE was adequate to meet the requirement of quality assurance for the clinical assessment?

**Quality as perfection or consistency:**

1. What aspects of the vOSCE did not work well, or requires further improvement?
2. What aspects of the vQA did not work well, or requires further refinement?

**Quality as fitness for purpose:**

1. Would you say the vOSCE and/or vQA are fit for purpose (meet requirements, fulfils stated objectives and mission, cost, technology, time, customer satisfaction)?

**Quality as value for money:**

1. Is the vOSCE process cost effective (when compared to delivering a face-to-face OSCE)?
2. Is the vQA process cost effective?

**Quality as Transformation:**

1. Did the delivery of an vOSCE allow for an enhanced educational/assessment experience for all participants?
2. Did the vQA process enhance the educational/assessment experience of participants (ie host school)?
